# Gut-on-a-chip models for dissecting the gut microbiology and physiology

**DOI:** 10.1063/5.0126541

**Published:** 2023-02-28

**Authors:** Amin Valiei, Javad Aminian-Dehkordi, Mohammad R. K. Mofrad

**Affiliations:** 1 Molecular Cell Biomechanics Laboratory, Departments of Bioengineering and Mechanical Engineering, University of California, Berkeley, California 94720, USA; 2 Molecular Biophysics and Integrative Bioimaging Division, Lawrence Berkeley National Lab, Berkeley, California 94720, USA

## Abstract

Microfluidic technologies have been extensively investigated in recent years for developing organ-on-a-chip-devices as robust *in vitro* models aiming to recapitulate organ 3D topography and its physicochemical cues. Among these attempts, an important research front has focused on simulating the physiology of the gut, an organ with a distinct cellular composition featuring a plethora of microbial and human cells that mutually mediate critical body functions. This research has led to innovative approaches to model fluid flow, mechanical forces, and oxygen gradients, which are all important developmental cues of the gut physiological system. A myriad of studies has demonstrated that gut-on-a-chip models reinforce a prolonged coculture of microbiota and human cells with genotypic and phenotypic responses that closely mimic the *in vivo* data. Accordingly, the excellent organ mimicry offered by gut-on-a-chips has fueled numerous investigations on the clinical and industrial applications of these devices in recent years. In this review, we outline various gut-on-a-chip designs, particularly focusing on different configurations used to coculture the microbiome and various human intestinal cells. We then elaborate on different approaches that have been adopted to model key physiochemical stimuli and explore how these models have been beneficial to understanding gut pathophysiology and testing therapeutic interventions.

## I. INTRODUCTION

The human gut is one of the critical organs with important biological functions orchestrated by human intestinal cells and a rich microbial consortium, known as the gut microbiome.^1^ The most well-known function of the gut is the digestion of food and absorption of nutrients.^2^ Being equipped with special enzymatic capacity, intestinal enterocytes are able to digest a variety of compounds such as amino acids, peptides, polysaccharides, and xenobiotics,^3–5^ and benefiting from a unique metabolic diversity, the microbiome maximizes the digestion by breaking down complex carbohydrates and vitamins.^6^ In addition to food digestion, the gut and its microbiome have broad roles in health regulation and disease prevention.^7^ A notable example of such roles is microbial and epithelial barrier functions, which are essential for maintaining homeostasis, establishing a bidirectional relationship with immune cells, and protecting the body against pathogenic attacks.^8,9^ The disruption of the gut barrier has been linked to various diseases, including gastrointestinal (GI) diseases, such as inflammatory bowel disease (IBD) and colorectal cancer,^10,11^ in addition to non-GI diseases as versatile as cancer, diabetes, obesity, asthma, cardiovascular diseases, and respiratory infections such as COVID-19.^12–17^

The eminent role of the gut in health and diseases has aroused tremendous interest among the scientific community in deciphering the gut's physiological complexities over the past decades; however, one of the major stumbling blocks in these attempts has been the limitations of conventional experimental techniques.^18^ Among various methods, direct experimentation on human subjects has been proven to be difficult due to the inaccessibility of the GI tract, and imaging techniques such as colonoscopy have been invasive and lacked cellular-level resolution.^19–21^ Analyzing the human microbiome has been conducted by sequencing fecal samples, but these are not representative of the entire gut microbial population, as many bacteria live in hardly accessible zones in the form of surface-attached communities, termed biofilms, inside the intestine.^22–25^ Moreover, fecal samples provide little information about the spatial variation of microbiota across the intestine.^26^ Animal models benefit from less strict experimentation protocols compared to humans, but they have major disadvantages due to their high cost, ethical issues, and the lack of high-quality real-time visualization capabilities.^27–30^ Most importantly, animals differ from humans in various physiological aspects such as their microbiome composition.^28,31^

Alternative to *in vivo* approaches has been *in vitro* strategies that have been developed to build organ mimicries that can be conveniently investigated in the lab in cheaper and more accessible manners.^32,33^ The conventional form of *in vitro* models has been 2D models such as well-plates and Petri dishes, whereby host cells and bacteria are cocultured on a planar surface.^34,35^ These models, however, have shown poor correlation to real tissue physiology due to their planar geometry.^34,35^ An improvement to the 2D *in vitro* modeling has been 3D organoids, which leverage the differentiation and self-organization ability of stem cells to construct organ-mimicking tissues.^36^ Organoids, nevertheless, are close-shaped structures and incompatible with modeling transport processes and the organ's internal environmental cues;^37,38^ accordingly, they have yet been unable to mimic complex tissue functions.^36^

Despite the limitations of conventional approaches, an alternative *in vitro* platform, proposed in recent years, is based on the organ-on-a-chip technology. Drawing on microfluidic capabilities, the organ-on-a-chip enables the simulation of 3D tissue geometries as well as the chemical, hydrodynamic, and mechanical cues of native organs, thus conceiving breakthrough avenues for modeling various organ functions.^39,40^ The organ-on-a-chip is currently considered one of the promising developmental fronts in *in vitro* biomedical research and has shown transformative prospects for modeling gut physiology.^41,42^ Considering the significance of this research field, the current review is devoted to a survey of the most current gut-on-a-chip achievements. The rest of this article is organized as follows: Sec. II examines the concept of organ-on-a-chip and how this new paradigm has been applied to simulate the gut with its unique physiology and structure; a breakdown of gut cellular models and various device configurations so far adopted has been presented. Section III offers an overview of the results obtained on modeling the fluid flow, peristalsis, and oxygen gradient in gut-on-a-chip models and how incorporating these cues can provide advantages over traditional static cultures. In Sec. IV, we present a detailed discussion of the utility of the gut-on-a-chip to model diseases and test drugs, the status of the translational efforts, and the concept of multi-organ-on-a-chip. Finally, the limitations of the gut-on-a-chip are outlined in Sec. V with the hope to inspire future research directions.

## II. THE ON-CHIP IMPLEMENTATION OF GUT PHYSIOLOGY

### A. The organ-on-a-chip concept

**FIG. 1.**
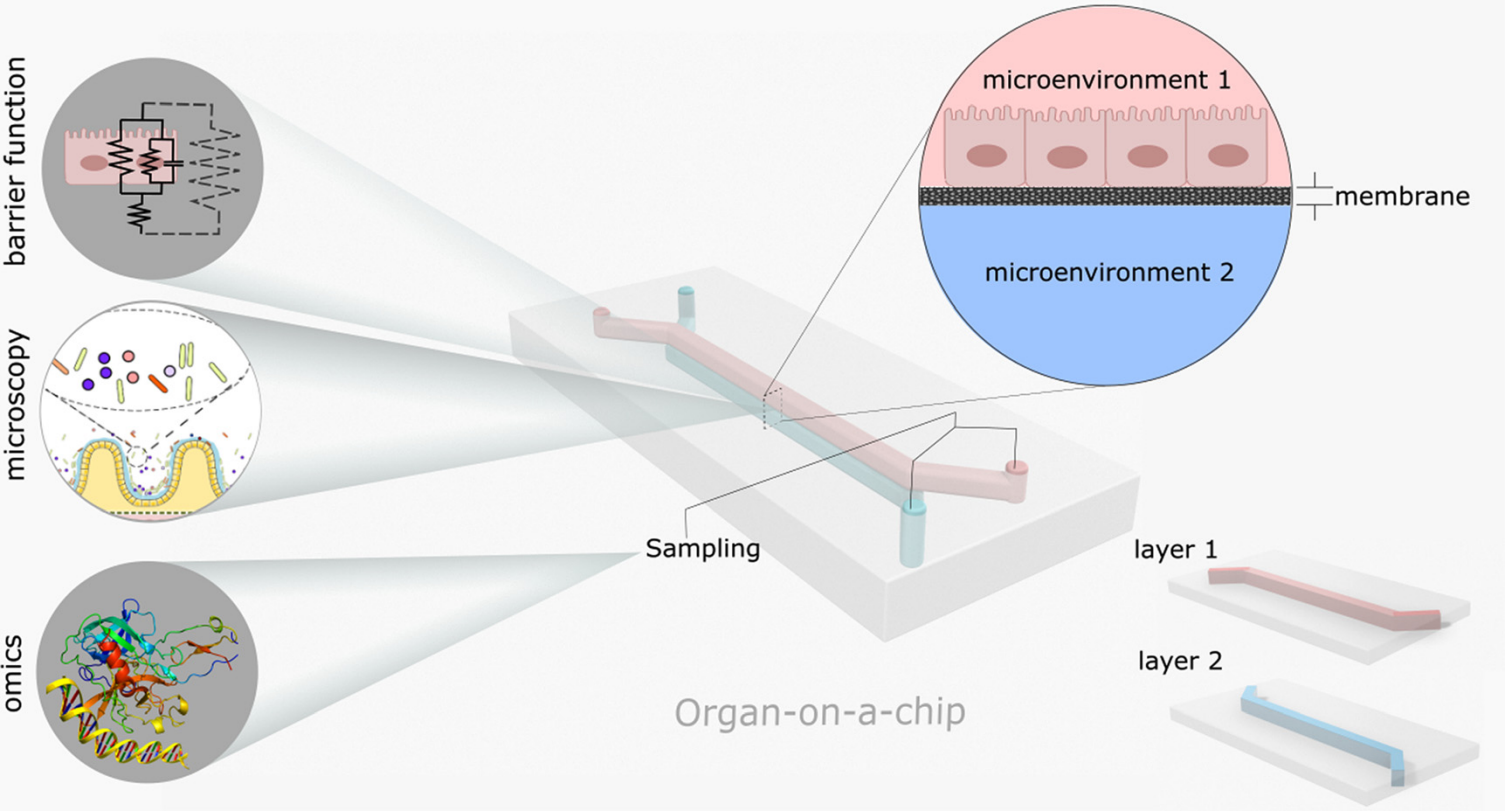
Schematic of a double layer organ-on-a-chip device. Each layer is built separately simulating a cellular microenvironment. A porous membrane can be used between the layers to create tissue interfaces across different microenvironments. Various analytical techniques such as microscopy as well as omics and histological analyses can be integrated with the devices to study biological processes (refer to Sec. II D for an explanation of analytical techniques).

Organ-on-a-chip takes advantage of a unique reductionistic approach to model human organs, breaking down a complex organ into its key constituent cellular microenvironments.^43,44^ The cellular microenvironment is the distinctive environment around each cellular entity that is essential for its development and growth.^39,45^ In organ-on-a-chip designs, microenvironments are typically simulated in a microfluidic module, often involving microchannels,^38,39^ which, owing to the laminar flow on small scales, makes it possible to control flow and transport processes such as chemical diffusion.^39,46^ Microchannels can be constructed in separate layers using microfabrication techniques, particularly soft lithography in which a polymeric material (e.g., polydimethylsiloxane, PDMS) is cast into a master mold.^39,47^ The master mold, which is fabricated by etching silicon or 3D printing, contains the negative replica of the desired microchannel pattern.^39,48^ Porous membranes, which could also be synthesized by polymer fabrication methods, can create interfaces between channels.^49^ The device components including fabricated PDMS layers and the membrane(s) are then bonded to form a 3D construct that has punched ports for fluid delivery and removal in each compartment (Fig. 1).^49^ Tissues are cultured in microfluidic channels after the device surface is treated with a proper coating material such as an extracellular matrix (ECM) scaffold.^49^ The porous membranes between microchannels interconnect the microenvironments and allow the passage of chemicals, completing the 3D organ milieu.^38,49^ The final organ models are integrated with fluidic and analytical devices to operate and record biological parameters (Fig. 1). The organ-on-a-chip approach has so far resulted in models of lung, liver, kidney, brain, heart, and other organs.^50–54^

### B. Intestine cellular models

The gut is an organ with a diverse cellular makeup with each cell type living in its specific microenvironment (see Box I for a review of gut physiology), which indicates the significance of compartmentation offered in organ-on-a-chip platforms in modeling the gut. The first step to realize a gut-on-a-chip is the selection and culture of appropriate cellular models. For microbiome studies, samples can be obtained directly from fecal samples, to be used freshly, or after preservation in animals (e.g., mice)^55^ or growth in *ex vivo* cultures (e.g., in SHIME^®^ reactors).^56^ Tissue models can be generated through a variety of methods. The primary cell culture is a direct culture technique; however, the resulting intestinal tissue is typically short-lived and unstable.^57,58^ A more popular type of cellular model consists of immortalized cell lines, which rely on the indefinite differentiation capability of tumor cells.^59^ A well-known immortalized cell line is Caco-2, which was obtained from human colon adenocarcinoma and reproduces several key intestine epithelial features such as tight junctions and brush borders^60,61^ (other model cell lines are T84 and HT-29^62^). The drawbacks of cancerous cells, however, are genetic and phenotypic aberrations and poor cytodifferentiation and tissue morphology^63–66^—as will be discussed later, these aspects can be improved by using organ-on-a-chip systems. Alternatively, adult stem cells, separated from biopsies, can be used to produce intestinal organoids, which could then seed new tissue cultures.^59,67^ The advantage of these cells is that they can be extracted from different intestine sections and grown into polarized and differentiated epithelial cell lineages.^68^ Intestinal organoids can also be acquired from pluripotent stem cells, by either reprogramming adult stem cells of various organs or from embryonic stem cells,^37,68^ thus circumventing the invasive deep-body biopsies. In addition to epithelium, various cell models are available for endothelial cells such as human umbilical vein endothelial cells (HUVECs) or human intestinal microvascular endothelial cells (HIMECs), and for immune cells such as human peripheral blood mononuclear cells (PBMCs).^69–71^ The existing cellular models provide flexibility to simulate cellular systems from the lumen down to the subepithelial tissue while providing means for validating and comparing the organ-on-a-chip against other *in vitro* approaches.

**FIG. 2.**
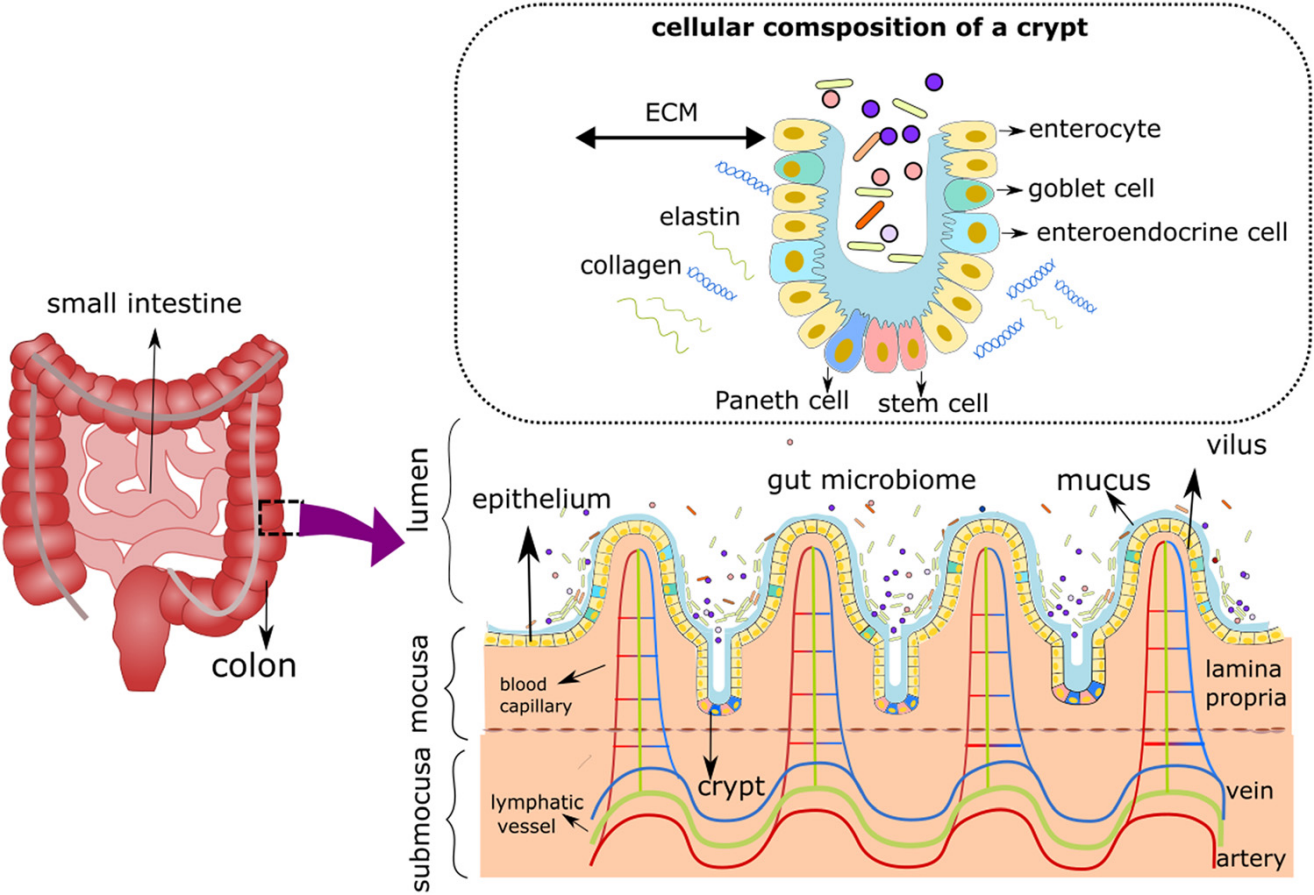
Schematics of the gut cellular composition and structure. The microbiota lives in the lumen, which features an anaerobic condition and a convective fluid flow. Epithelial cells surround the lumen's outer surface and form 3D villi and crypts (the inset shows the cellular composition of a crypt as described in Box I). Below the lumen is the mucosa, which embeds aerobic blood and lymph arteries in lamina propria; this area is populated by a diverse cellular makeup ranging from endothelial cells to immune cells.

**Box I.** Intestine cellular physiology.

A brief review of the gut physiology is provided here with specific focus on the important role of cellular microenvironment in organ-on-a-chip systems. The gut possesses an immensely diverse cellular system spanning different anatomical sections, including the lumen, epithelium, and sub-epithelium (Fig. 2). The microbiome consists of hundreds of species of bacteria, viruses, yeasts, and fungi that live in the lumen under the condition of the convective flow of the digested food and the fluctuating mechanical motion, termed peristalsis, that propels the food forward.^72–74^ Lumen is characterized by an anaerobic environment, resulting from the oxygen consumption of microbes during food digestion.^74,75^ The environmental variables themselves vary widely along the lumen; for example, the colon features a slower flow and less acidic pH compared to the small intestine, causing a denser microbial community.^26,76^ Encircling the lumen are epithelial cells, a monolayer of closely adjoined cells shaped naturally into a 3D topography, consisting of villi (peaks) and crypts (valleys).^77^ The bottom of the crypt is made of stem cells performing tissue renewal during which most differentiated cells migrate toward villi.^78^ The differentiated epithelial lineages include columnar enterocytes (absorptive cells), which are responsible for food absorption, goblet cells, which produce a protective mucin layer, Paneth cells, which excrete antimicrobial peptides, and endocrine cells, which release gastrointestinal hormones.^77^ Besides these roles, the tight junctions provided by the epithelium provide structural integrity for the gut tissue.^67^ Beneath the epithelium is a niche consisting of structural and cellular components that are essential for maintaining its homeostasis.^79^ The underlying ECM provides physical support for the epithelium and contains a structured network of collagen fibers, integrins, fibronectin filaments, laminins, and glycosaminoglycan.^79^ The cellular components in this area, termed lamina propria, are diverse comprising structural elements (fibroblasts, fibrocytes, and vascular endothelial cells) in addition to blood and immune cells.^80^ Lamina propria is nutritious, oxygenated, and rich in blood and lymphatic capillaries, which carry the absorbed nutrients off the gut.^67,81^ Overall, the structure of the gut cellular system varies widely in terms of composition, architecture, physicochemical cues, and function across the organ's anatomy. It is clear that without a holistic approach to model cellular microenvironments, it is impossible to capture the physiological and functional complexity of the gut.

### C. Microfluidic designs

Considering the multiplicity of cellular environments and the availability of various cellular models, several microfluidic designs have been thus far devised to study the gut. Depending on the complexity of design philosophies, the models can be broadly divided into mono-environment and multi-environment designs (refer to Table I for a summary of key models).

**TABLE I.** Microfluidic platforms for gut physiological modeling.

Authors	Device name and structure	Flow (normal operation)	Peristalsis^d^	Aerobics	Human cells	Microbes	Experimental time^a^
Cremer *et al.*^76^	The minigut: single minichannel—lumen (microbes)	0–50 *μ*m s^−1^	Consecutive valve actuation along the channel	Aerobic	NA	*E. coli*	20 h
Guo *et al.*^83^	Microchannels simulating lumen (with a porous membrane)	400 *μ*l h^−1^	No	Aerobic	Caco-2	NA	5 days (D) (+12 h drug test)
Fois *et al.*^82^	Single microchannel—lumen	29 *μ*l h^−1^ (Normal)	No	Aerobic	Caco-2 HTB-37™	NA	8 D
Chi *et al.*^109^	*μ*FCCD: 2 stacked microchannels—an upper (enterocytes) and a lower channel	0.5 *μ*l min^−1^	No	Aerobic	Caco-2	*Salmonella enterica* S. Typhimurium	3 D
Secchi *et al.*^84^^,^^b^	Microchannels with pilllars or corrugated topography	0.6–6 *μ*l min^−1^	No	Aerobic	NA	*P. aeruginosa* PA14	5 h
Kim *et al.*^85^^,^^b^	Microchannels with crevices	0.1–100 *μ*l min^–1^	No	Aerobic	NA	*S. aureus, V. cholerae*	∼several hours (e.g., up to 30 h)
Marzorati *et al.*^86^	HMI: two stacked microchannels—lumen (microbes), and host (enterocytes)	Lumen: 6.5 ml min^−1^, host: 2 ml min^−1^	No	Aerobic lower chamber and anaerobic upper chamber	Caco-2	Complex microbial community derived from SHIME^®^ reactors (*Lactobacillus rhamnosus* GG as the control)	7 D human cells, 2 D coculture
Shah *et al.*^87^	HuMiX: three stacked microchannels—lumen (microbes), human chamber (epithelial), and perfusion chamber	Lumen: 25 *μ*l min^−1^, perfusion chamber: 25 *μ*l min^−1^	No	Aerobic perfusion chamber and anerobic microbial suspension	Caco-2 or CCD-18Co (human chamber), CD4^+^ T in the perfusion chamber^c^	*Lactobacillus rhamnosus* GG (LGG), *Bacteroides caccae*	7 D human cells, 1 D coculture
Kim *et al.*^88^	Two stacked microchannels—lumen (epithelial, microbial cells) and a lower channel	Both channels 30–40 *μ*l h^−1^	Lateral vacuum chambers 10% strain, 0.15 Hz	Aerobic	Caco-2	*Lactobacillus rhamnosus* GG (LGG)	∼4–5 D human cells, >1 week coculture
Kim *et al.*^94^	Two stacked microchannels—lumen (epithelial, microbial cells) and vascular	Lumen: 30 *μ*l h^−1^, vascular: 30 *μ*l h^−1^	Lateral vacuum chambers 10% strain, 0.15 Hz	Aerobic	Caco-2, ±PBMCs	A select mixture of gut microbes	∼100 h human cells, 72 h coculture
Kasendra *et al.*^71^	Duodenum intestine-chip: two stacked microchannels—lumen (epithelium) and vascular	Lumen: 60 *μ*l h^−1^, vascular: 60 *μ*l h^−1^	Lateral vacuum chambers 10% strain, 0.2 Hz	Aerobic	Biopsy-derived organoids (lumen), ±HIMECs (vascular)	NA	12 D
Workman *et al.*^93^	Two stacked microchannels—lumen (epithelium) and vascular	30 *μ*l h^−1^	Lateral vacuum chambers 10% strain; 0.2 Hz	Aerobic	Dissociated organoids generated from induced pluripotent stem cells (CS83iCTR-33n1 and CS688iCTR-n5)	NA	∼2 Weeks
Jalili-Firoozinezhad *et al.*^55^	Two stacked microchannels—lumen (epithelium) and vascular	Lumen: 60 *μ*l h^−1^, vascular: 60 *μ*l h^−1^	Lateral vacuum chambers 10% cell strain, 0.15 Hz frequency)	Anerobic (upper chamber), aerobic (bottom suspension)	Human intestinal organoids (lumen), HIMECs (vascular)	*B. fragilis*, human microbiota colonized in mice, microbiota derived from fecal samples	∼1 Week human cells, 5D coculture
Shim *et al.*^95^	Two stacked microchannels—apical and basolateral chambers integrated with a 3D collagen scaffold	100 *μ*l min^−1^	No	Aerobic	Caco-2	NA	∼14 D
Shin *et al.*^96^	Two stacked microchannels—lumen and vascular channels	Lumen 50 *μ*l h^−1^, vascular 50 *μ*l h^−1^	Lateral vacuum chambers 5% average elongation at 0.15 Hz	Anerobic microbial culture	Biopsy-derived organoids from patients with GI diseases (lumen) or Caco-2 cells (lumen)	Fecal samples	7–10 D human cells, 2 D coculture
Jing *et al.*^97^	Three layers, a central lumen and two surrounding vascular channels	Vascular: 60 *μ*l h^−1^, lumen: 0–85 *μ*l h^−1^	Peristaltic flow induced by a pump in the lumen channel (0. 15 Hz)	Aerobic	Caco-2 (lumen), HUVEC (vascular),^c^ macrophages U937 (vascular)^c^	*E. coli, L. casei*	∼5 D human cells, 7 D coculture

^a^ The experimental time refers to an example time frame where the device has been reported to stay functional (they might support longer times). Measurements could be collected at earlier times.

^b^ These studies are not explicitly designed as gut-on-a-chips but focus on microfluidic modeling of bacterial attachment on 3D villi and crypt-like topographies.

^c^ Refers to the optional cell cultures.

^d^ The use of peristaltic pumps to drive liquids in the channels was not considered a model of intestinal peristalsis unless engineered and systematically studied for that purpose.

#### 1. Mono-environment models of the gut

**FIG. 3.**
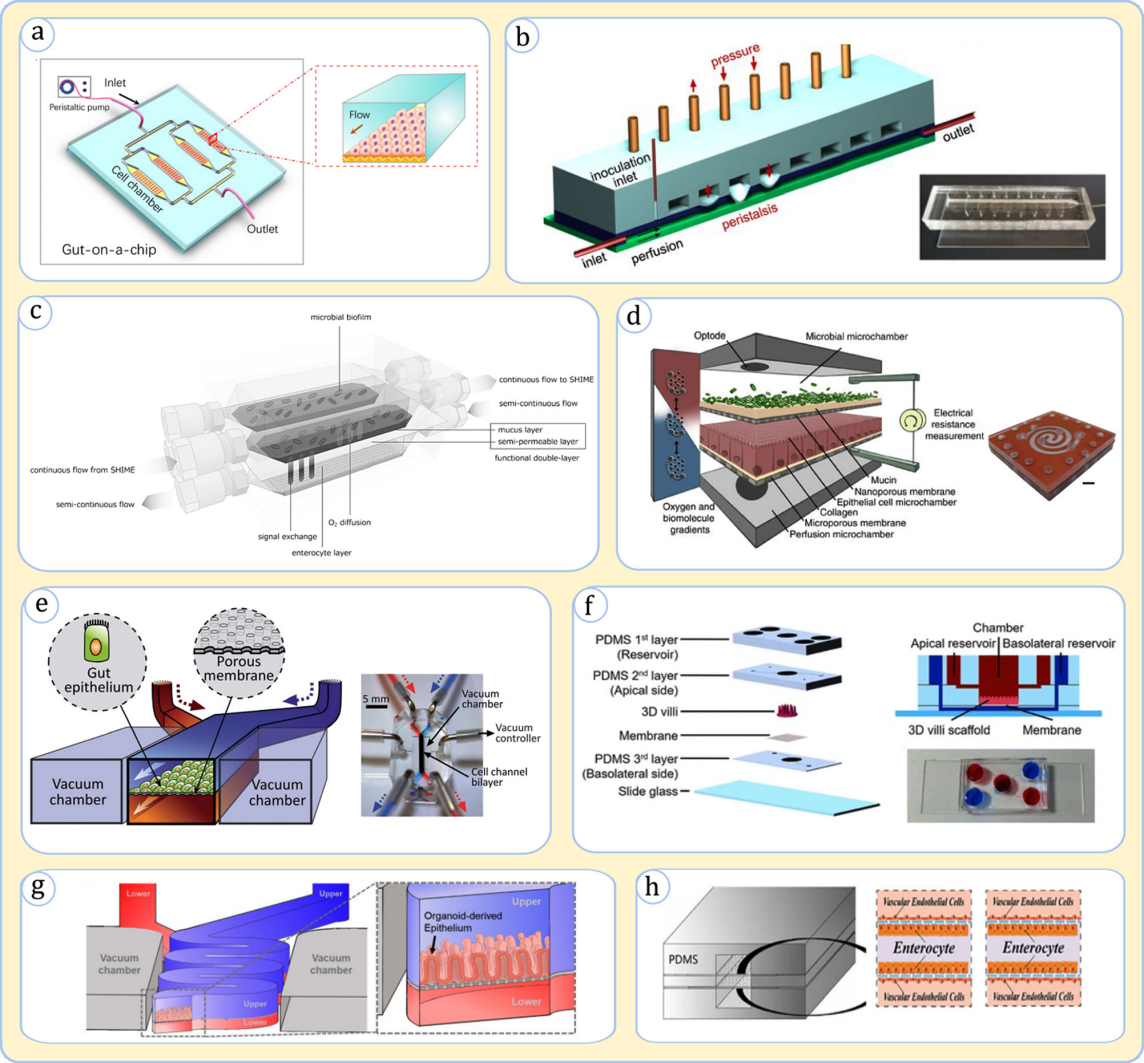
Representative gut-on-a-chip designs. (a) A device with microchannels to culture intestinal cells. Adapted with permission from Guo *et al.*, Artif. Organs **42**(12), 1196 (2018). Copyright 2018 John Wiley and Sons.^83^ (b) The minigut consisting of a microchannel with an air valve array producing programmed deformations similar to peristalsis. Adapted with permission from Cremer *et al.*, Proc. Natl. Acad. Sci. **113**(41), 11414 (2016)^76^ (the photo of the chip at the lower right was obtained from the supporting information of the article). (c) HMI: a device containing one channel for the microbiome and one for enterocytes. Adapted with permission from Marzorati *et al.*, BMC Microbiol. **14**(1), 133 (2014). Copyright 2014 Authors, licensed under a Creative Commons Attribution (CC BY) license.^86^ (d) HuMiX: a microfluidic device that simulates microbiome, epithelium, and subepithelial perfusion in separate channels. From Shah *et al.*, Nat. Commun. **7**(1), 11535 (2016). Copyright 2014 Authors, licensed under a Creative Commons Attribution (CC BY) license.^87^ (e) A device with a luminal channel, containing both the microbiome and enterocytes, and a parallel vascular channel. The device embeds lateral vacuum chambers to generate peristalsis. Used with permission from Kim *et al.*, Lab Chip **12**(12), 2165 (2012). Copyright 2012 Clearance Center, Inc.^88^ (f) A device with luminal and vascular microchannels interfaced by a 3D villi collagen scaffold. Adapted with permission from Shim *et al.*, Biomed. Microdevices **19**(2), 37 (2017). Copyright 2017 Springer Nature.^95^ (g) A device with luminal and vascular microchannels in a convoluted shape. Adapted with permission from Shin *et al.*, Micromachines **11**(7), 663 (2020). Copyright 2020 Authors, licensed under a Creative Commons Attribution (CC BY) license.^96^ (h) A device with a central luminal channel flanked by two vascular channels. The flow in the lumen is driven by peristalsis. Adapted with permission from Jing *et al.*, Front. Bioeng. Biotechnol. **8**, 272 (2020). Copyright 2020 Authors, licensed under a Creative Commons Attribution (CC BY) license.^97^

The simplest form of gut microfluidic models has been devices that mimic a single type of microenvironment and typically culture either microbial or intestinal cells. These devices have easily mimicked *in vivo* fluidic transport and shear forces. A simple implementation of this model is a device that hosts Caco-2 cells under the lumen hydrodynamics in a microchannel [Fig. 3(a)], which was used in several studies for gut physiological investigations.^82,83^ The simple channel model could be further modified to simulate environmental cues such as gut peristalsis. For example, the “minigut” device developed by Cremer *et al.* to study bacterial growth dynamics [Fig. 3(b)] modeled intestinal wall contractions through successive pressure-actuated membrane valves along the channel.^76^ In microbial studies, micro topographies have been embedded in various studies to simulate the gut epithelial villi topography, capturing microscale cell-surface interactions.^84,85^ Mono-environment devices offer simple practical means for modeling biological processes in a specific niche; however, they often disregard the “big picture” interplay between different microenvironments.

#### 2. Multi-environment gut-on-a-chip devices

In situations demanding more rigorous analysis of gut physiology, multi-environment gut-on-a-chip devices have undeniably been preferred. They have been typically composed of multi-layer devices with stacked interconnected microchannels. Here, we discuss some of the major schemes developed thus far (Table I).

##### a. HMI (host–microbiota interaction) and HuMiX (human–microbial crosstalk):

One of the highlighted design approaches for the gut-on-a-chip has been a scheme in which separate channels are allocated to microbial and intestinal cell environments. A pioneering device in this category has been the HMI platform proposed by Marzorati *et al.* [Fig. 3(c)].^86^ HMI contains a microbial and a Caco-2 cell culture channel partitioned by a microporous membrane with a deposited mucus layer. The segregated configuration of HMI has shown great advantage in reducing the cytotoxicity of a complex microbiome population on intestinal cells.^86^ The device supported a viable coculture for multiple days following more than a week of stand-alone tissue culture.^86^ Another example in this category has been a three-channel design, termed HuMiX, developed by Shah *et al.*^87^ This device expanded on the HMI concept by simulating the basal side of epithelial Caco-2 cells, in a separate microchannel, termed perfusion chamber, and adding a porous membrane between the perfusion chamber and epithelial chamber on which epithelial cells were cultured [Fig. 3(d)].^87^ Various assays demonstrated the HuMiX's ability to hold viable microbiota and intestine tissue cells mimicking numerous *in vivo* cellular signatures.^87^

##### b. Emulate

A different gut-on-a-chip design approach features two separate channels for modeling the vascular and luminal environments separated by a microporous membrane on which epithelial cells are cultured [Fig. 3(e)].^88^ In this device, the microbial cells are injected into the lumen to be in contact with the epithelium being reminiscent of their real-life intimate arrangement.^88^ This device design, which was pioneered by Ingber's group^88^ and is being manufactured commercially by Emulate, Inc., has been used in numerous investigations.^89–91^ The device in its typical configuration harbors chambers on the lateral sides of the microchannels to induce fluctuating vacuum that emulates intestinal peristalsis.^88^ This platform has been shown to stimulate key phenotypic changes in the epithelium, such as the spontaneous development of villi topographies and the secretion of mucin.^88,92^ The device was shown to maintain an effective viable tissue barrier over a few weeks in cultures of either intestinal cancer cell lines or organoid-derived cells,^71,88,93^ and for at least several days in cocultures with the microbiome.^55^ In some variations, the device has embedded endothelial and immune cells in the bottom channel to recapitulate the subepithelial zone.^71,94^

##### c. Other designs

Other creative designs have also been developed building on earlier models. Shim *et al.* developed a platform that incorporated a two-microchannel arrangement to simulate luminal and vascular microenvironments, but, as a new feature, they embedded a villi-like collagen scaffold on the dividing porous membrane to promote the tissue topography [Fig. 3(f)],^95^ which resulted in a valid model surviving for at least two weeks. A different design by Shin *et al.* was similar to Emulate platform but used a convoluted channel in an attempt to more realistically model the fluid flow and to improve the culture residence time [Fig. 3(g)].^96^ In another design, Jing *et al.* aimed to capture the symmetrical geometry of the gut by building a central lumen for coculturing microbial and epithelial cells with two surrounding vascular channels [Fig. 3(h)].^97^ This model, in which the luminal flow is driven by a peristaltic pump, managed to maintain a viable culture of the gut tissue for at least one week.^97^

### D. Analytical measurements

The modular structure of organ-on-a-chip devices, be it mono- or multi-environment, enables each compartment to be integrated with various analytical techniques (Fig. 1). Being fabricated using transparent materials such as glass and PDMS, high-resolution visualization techniques, like confocal laser scanning microscopy and phase contrast microscopy, allow for *in situ* monitoring of cells and tissues.^98^ Fluorescent and immunofluorescent staining of biomolecules can yield critical information on cell viability and cellular functions,^98^ and cellular signatures can be obtained by omics approaches such as genomics, proteomics, transcriptomics, and metabolomics from microchannels.^87,99^ Such data can be utilized to highlight physiological changes in each compartment. Furthermore, the combination of fluorescence microscopy with DNA probes can be used to perform genetic and transcriptomic mapping of cells and tissues using the fluorescence *in situ* hybridization (FISH) assay.^100^

Considering the critical barrier role of the gut, barrier integrity assays can be conveniently incorporated into microchips.^101–103^ These techniques include qualitative methods such as fluorescence immunostaining of tight junctions or quantitative ones such as permeability assessments by injection of fluorescent-labeled molecules or transepithelial electrical resistance (TEER) measurements.^101–103^ TEER, which measures ionic conductance across a tissue layer, has particularly been highlighted due to its non-invasiveness and high accuracy.^104^ In gut on-a-chip platforms featuring apical and basal channels, TEER measurements were performed across epithelium cultured on a porous membrane to assess tissue integrity (TEER of ∼4000 Ω cm^2^ reported by Kim *et al.*^88^ and ∼1000 Ω cm^2^ by Shah *et al.*^87^ were substantially higher than values reported in planar static cultures; for a detailed discussion of TEER, refer to the review by Srinivasan *et al.*^101^).

## III. GUT-ON-A-CHIP MERITS FOR SIMULATING ENVIRONMENTAL CUES

Gut-on-a-chip designs have so far appeared in different platforms and have exhibited remarkable advantages over conventional counterparts for simulating fluid flow, mechanical forces, and oxygenation conditions (Table I lists the description of these parameters for various devices). Accordingly, gut-on-a-chip research, above everything, has provided new understanding of how these factors influence gut physiology. Herein, we touch on insights that different gut-on-a-chip models have given by their unique approach.

### A. The effect of fluid flow

**FIG. 4.**
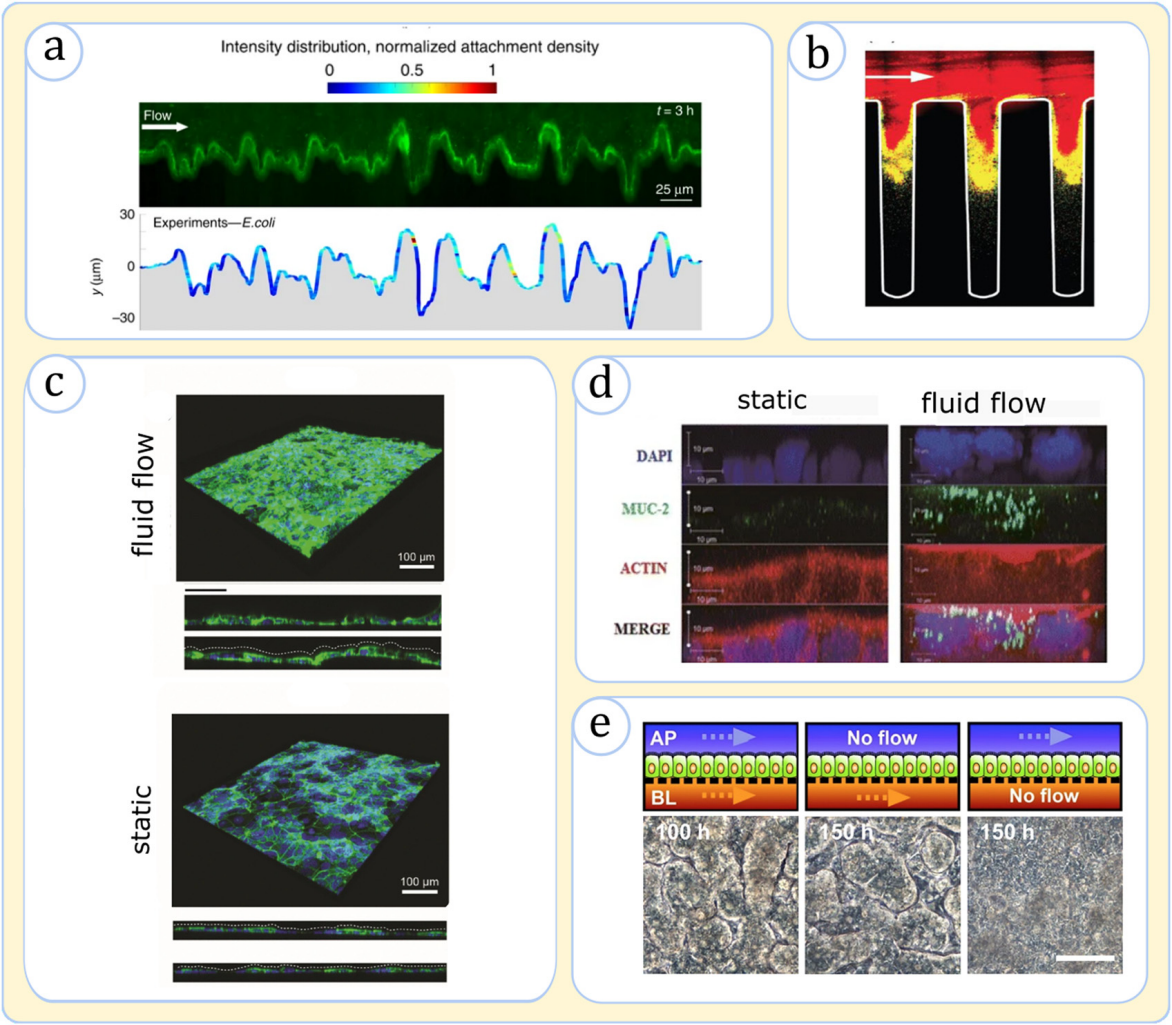
Physiological effects of hydrodynamics in microchips. (a) Fluorescence image showing the distribution of GFP-tagged *E. coli* (green) on a corrugated topography exposed to fluid flow. The variation of shear causes higher cellular attachment at the leeward face of the protrusions. Adapted from Secchi *et al.*, Nat. Commun. **11**, 2851 (2020). Copyright 2020 Authors, licensed under a Creative Commons Attribution (CC BY) license.^84^ (b) Images of *S. aureus* biofilms in a complex topography in a microfluidic channel. The presence of fluidic streams washes away the signaling molecules, allowing the quorum sensing (QS) to occur mostly within the crevices (red shows QS-off cells). Adapted with permission from Kim *et al.*, Nat. Microbiol. **1**, 15005 (2016). Copyright 2016 Springer Nature.^85^ (c) Fluorescence images of Caco-2 tissue in a microfluidic channel upon exposure to dynamic flow and in a static culture, both after 8 days of culture. The fluidic shear promotes the expression of F-actin (green), the filamentous structural support for microvilli, and a 3D morphology resembling *in vivo* tissue undulations (compare the side views). Adapted with permission from Fois *et al.*, Biomed. Microdevices **23**(4), 55 (2021). Copyright 2021 Authors, licensed under a Creative Commons Attribution (CC BY) license.^82^ (d) Side-view images of epithelial cells upon exposure to flow for 3 days and static Transwell after 21 days. The tissue is stained with MUC-2 antibody (green), actin (red), and 4′,6-diamidino-2-phenylindole (DAPI) (blue). The flow condition causes faster morphogenesis and higher mucin secretion. Adapted with permission from Chi *et al.*, Biomed. Microdevices **17**(3), 9966 (2015). Copyright 2015 Springer Nature.^109^ (e) Top-view images of the epithelium illustrating the effect of apical and basolateral flow in the morphogenesis of the cultured epithelial cells. A parallel flow on both sides of the epithelium (or at least the bottom channel) is needed to induce undulating tissue morphology. Adapted with permission from Shin *et al.*, iScience **15**, 391 (2019). Copyright 2019 Elsevier^108^ (top sketches show the side-views of the device configuration).

An indispensable advantage of using microchip systems has been recapitulating gut hydrodynamic conditions. Various mono-environment devices demonstrated that hydrodynamics significantly impacts the biological behaviors of microbial and intestinal cells. Using the minigut concept, Cremer *et al.* showed that the luminal fluid flow is a key determiner of bacterial growth kinetics, affecting the rate of metabolic processes and the spatiotemporal distribution of bacteria.^76^ Secchi *et al.*,^84^ Valiei *et al.*,^105^ and Jahed *et al.*^106^ indicated that fluid shear on posts and villi-like topographies can significantly impact bacterial interactions with these surfaces [Fig. 4(a)]. Notably, these researchers discovered that shear force variation strongly influences bacterial attachment,^84^ which, in the latter two works, was shown to instigate the formation of unconventional bacterial structures such as streamers and networks.^105,106^ Kim *et al.* revealed that the fluidic fields on corrugated topography strongly impact bacterial collective behaviors such as quorum sensing (QS) [Fig. 4(b)]^85^ as fluid-protected niches, particularly the cavities inside crypts, create appropriate spots for the buildup of signaling molecules, promoting the formation of bacterial biofilms—this underscores the *in vivo* observations of crypts' pathogenic invasions.^107^

Fluid flow affects intestinal cells through various mechanisms. Interestingly, reports unveiled enterocytes exposed to flow reproduce villi-like and microvilli architectures observed in the natural tissue, whereas those features were lacking under a static culture [Fig. 4(c)].^82^ Moreover, the dynamic flow initiated important physiological effects such as mucin secretion, cell polarization, and the formation of tight junctions [Figs. 4(c) and 4(d)].^71,82,88,108^ Multi-environment devices simulating both apical and basal environments around the epithelium (as in the Emulate device) could better emulate the physiological effect of fluid flow.^82,88,95,97^ Compared to static conditions, such bilateral flows better replicated the barrier permeability and phenotypic characteristics in both Caco-2 and organoid-derived culture models.^71,88^ Intriguingly, while Caco-2 cells had been traditionally known to grow to enterocyte-like phenotypes, under a parallel microfluidic flow, they spontaneously differentiated into multiple lineages (absorptive, enteroendocrine, Paneth, and goblet cells) and underwent significant morphogenesis to form tall villi-like formations.^71,88,90,92^ The morphogenesis has been recently attributed to the hydrodynamic removal of signaling molecules (particularly Wnt) from the basolateral side of the tissue, which could hint at a new physiological mechanism in the intestine development [Fig. 4(e)].^71,108^

Finally, fluid flow has been found to be a crucial factor in modulating effective host-microbial interplay. Static cocultures have been shown to cause inharmonious growth of cells and the accumulation of undesirable cellular metabolites, eventually causing the death of epithelial cells and microbial overgrowth.^88^ In contrast, the luminal fluid has been essential to extend a viable culture to a few weeks, much longer than cell longevities in Transwells.^55,87,88^ Overall, previous works have unanimously demonstrated that fluid flow is an indispensable element in gut physiology without which a viable model of the human intestine is impossible.

### B. Addition of cyclic motion

The ability to create cyclic motion is another unique addition to the gut-on-a-chip systems. The mechanical peristaltic motion, whether originating from gas-actuated channels or fluctuating pumps, has been shown to stimulate important physiological effects.^88,97^ The minigut model demonstrated that intestinal peristalsis is responsible for mixing the food and microbial population, which is necessary to achieve efficient metabolism.^76^ The lack of peristalsis, conversely, resulted in stratified flow leading to instability and microbial washout.^76^ Peristalsis's effects on intestinal cells have been identified in a few studies, uncovering effects such as the elevated expression of key enzymes, including cytochrome P450 3A4 (CYP3A4), a pivotal effector in drug metabolism, sucrase-isomaltose, a critical catalyst for sugar decomposition as well as villin, a functional protein found in brush border membranes.^71,92^ At the transcriptome level, peristalsis enhanced gene expressions related to ion, lipoprotein, and water transportation, as evidenced in the analysis of organoid-derived epithelial cells on chip.^110^

**FIG. 5.**
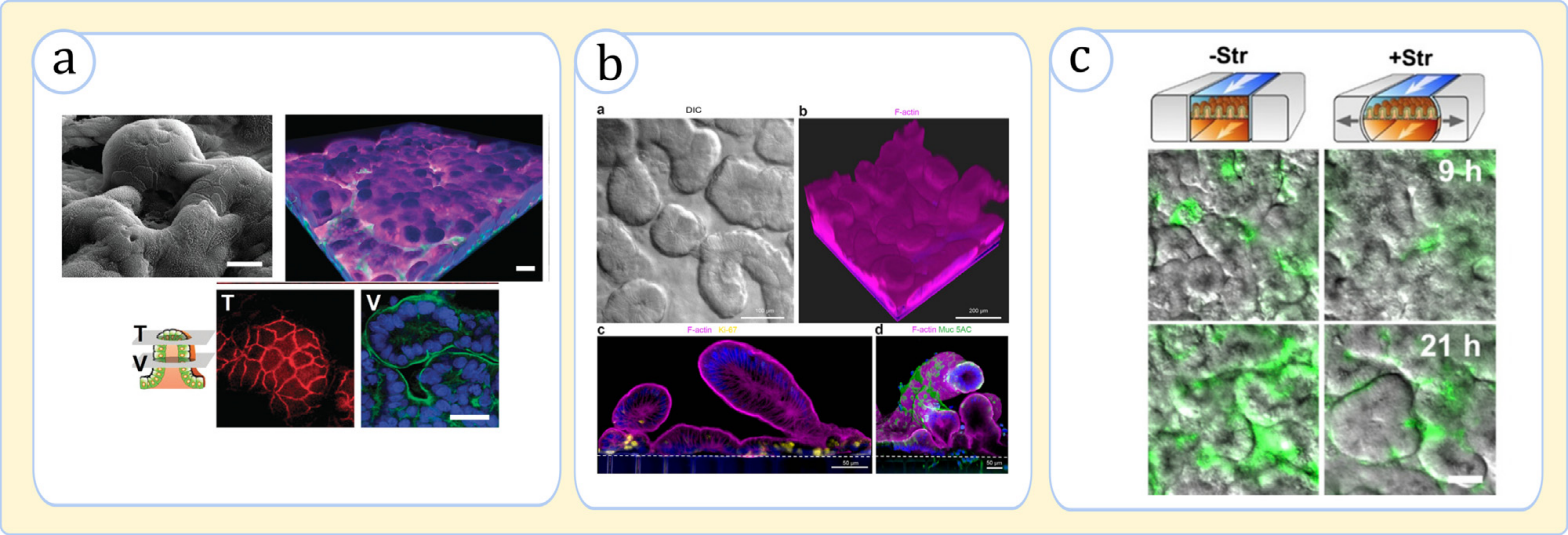
Physiological effects of cyclic mechanical forces in gut-on-a-chip devices. (a) Caco-2 cells under flow and cyclic mechanical stimuli form a well-defined polarized 3D topography, (top left) SEM image of villi, (top right) *Z*-stacked fluorescence image of villi (nuclei: blue, F-actin: green, and mucin 2: magenta), and (bottom row) images of a villus's top (T) and middle (V) cross sections (ZO-1 protein: red, F-actin: green). Adapted with permission from Kim *et al.*, Integr. Biol. **5**(9), 1130 (2013). Copyright 2013 Oxford University Press.^92^ (b) Organoid-derived epithelial cells form a villi-crypt topography in the presence of fluid flow and peristalsis (F-actin: magenta, Muc5AC: green, and nuclei: blue). Adapted with permission from Kasendra *et al.*, Sci. Rep. **8**(1), 2871 (2018). Copyright 2018 authors, licensed under a Creative Commons Attribution (CC BY) license.^71^ (c) The effect of pulsation on bacterial growth. The images (top-view of the epithelium) show the interaction of the *E. coli* bacteria (green) and villi topography (gray scale). The bacterial fluorescence intensity is much higher upon the loss of the mechanical strain (-Str), indicating significant overgrowth. Adapted with permission from Kim *et al.*, Proc. Natl. Acad. Sci. **113**(1), E7 (2016).^94^

In addition to its key role in digestion, chip models showed that mechanical pulsations induce important tissue responses.^88^ When *in vitro* tissue cultures were exposed to fluid flow and a cyclic mechanical strain, the epithelial permeability measurements revealed more physiologically relevant values than when they were only subjected to fluid flow.^88^ Moreover, these conditions improved cellular differentiation, structural mimicry of the villi-like formations [Figs. 5(a) and 5(b)],^97,108^ and the mucin production where the latter, in the case of organoid-derived cultures, recapitulated *in vivo* values.^71,88,90^ In addition, the peristalsis promoted glycocalyx secretion and microvilli formation in epithelial cells.^97^

In terms of host tissue–microbiome interaction, although the mechanistic impact of peristalsis is yet unclear, there is evidence to consider mechanical cues a potent factor in this interplay.^97^ Kim *et al.* observed that bacteria exposed to flow without peristalsis grow more than twofold faster than the bacteria exposed to simultaneous flow and peristaltic motion [Fig. 5(c)].^94^ This perfectly aligned with the observations that bacteria under a flow-peristalsis effect displayed substantially stronger enzymatic activity than static cultures.^88^ Altogether, previous investigations, in tandem, confirm that the implementation of mechanical cues guarantees a higher fidelity in the *in vitro* modeling of the intestine.

### C. Anaerobic condition

**FIG. 6.**
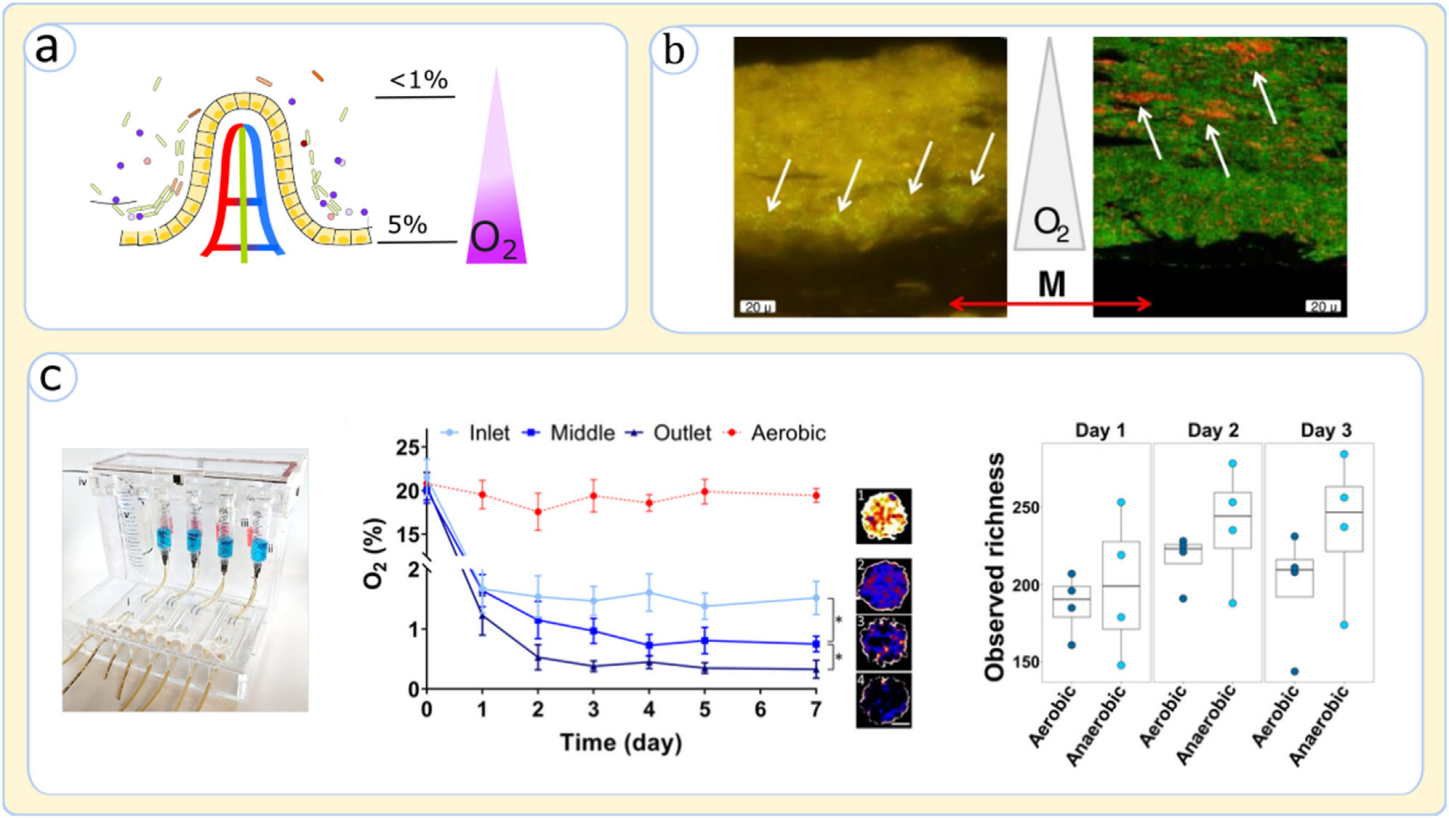
Effects of oxygenation control in gut-on-a-chip devices. (a) Oxygen gradient across the lumen-mucosa axis on a villus. The oxygen concentrations values are based on Singhal *et al.*^75^ (b) Effect of oxic conditions on the gut biofilm structure explored in HMI gut-on-a-chip by fluorescence *in situ* hybridization microscopy; (left) *F. prausnitzii* bacteria, marked with white arrows, have some tolerance to oxygen and tend to crowd in the lower side of the mucus layer; (right) *bifidobacterium* spp., as a strict anaerobe, marked by white arrows, is prominently observed at the upper side of the biofilm and the mucus layer (M shows the location of the membrane and mucus layer). Adapted from Marzorati *et al.*, BMC Microbiol. **14**, 133 (2014). Copyright 2014 Authors, licensed under a Creative Commons Attribution (CC BY) license.^86^ (c) The setup for the creation of an anaerobic environment in the Emulate gut-on-a-chip; (left) both the device and the culture for the epithelial channel are housed in an anaerobic atmosphere; (middle) the concentration of oxygen measured at the inlet, middle, and outlet of the epithelium-harboring channel after 7 days of culture; the *in vivo* oxygen concentration is almost achieved; (right) anaerobic culture of human stool samples creates a richer bacterial population than the aerobic condition. Adapted with permission from Jalili-Firoozinezhad *et al.*, Nat. Biomed. Eng. **3**(7), 520 (2019). Copyright 2019 Springer Nature^55^ (the image of the setup was obtained from the supplementary information of the article).

Although microfluidic devices that solely host intestinal cells are typically operated under aerobic culture media, microbial culture requires an anaerobic culture to grow in body-like conditions [Fig. 6(a)]. A few studies have so far tested devices with oxygen gradient across microfluidic layers by regulating the media flow and the oxygen concentration.^55,86,87^ HMI has been one of the successful platforms for creating differential oxygen level between epithelial cells and microbes, which illustrated that the spatial distribution of bacteria across the lumen and mucosa is heavily impacted by the oxygen availability [Fig. 6(b)].^86^ Specifically, the strict anaerobes showed a particular tendency to accumulate inside the lumen and the upper layer of the mucosal biofilm, whereas bacteria with more tolerance to oxygen were concentrated at the microaerophilic niche near the base of the microbial entities.^86^ Jalili-Firoozinezhad *et al.*, who incorporated an anaerobic enclosure to control the oxygen in the Emulate chip [Fig. 6(c)],^55^ discovered that alteration of oxygen influences the richness and abundance of bacteria. Their observations clearly underpinned the fact that microbiome models could only resemble *in viv*o compositions when living in a controlled anaerobic niche [Fig. 6(c)].^55^ Shah *et al.* further noted creating anaerobic conditions in the lumen substantially impacts the gut tissue physiology besides the microbiome.^87^ Investigating *Lactobacillus rhamnosus* cultures in HuMiX, they detected substantial differences in gene expression patterns for Caco-2 related to metabolism, cellular homeostasis, and interaction with the immune system.^87^ These studies suggest that the oxygen gradient across the gut tissue has profound impacts on cellular functions, substantiating that controlling oxic conditions can improve the mimicry of the microbiome and host interactions.

## IV. APPLICATIONS OF GUT-ON-A-CHIP DEVICES

The ability to produce hydrodynamical, mechanical, and chemical properties of the gut has made the gut-on-a-chip an excellent platform for launching medical and biological investigations. Gut-on-a-chip platforms have so far been used in important research domains, including the pathogenesis of diseases and therapeutic discoveries.

### A. Mechanistic insight into diseases (pathology)

Most GI diseases are challenging to study due to their convoluted links to a variety of environmental and genetic factors.^111^ Considering that animal models fall short to dissect etiological cause-effect relations due to inherent confounding effects present in complex organisms,^111^ gut-on-a-chip platforms, which have engineered simpler structures, have been sought as potential candidates to model multifactorial disease scenarios. In gut-on-a-chip systems, multiple cellular or environmental variables of relevance can be evaluated, and physiological effects derived from each modulation can be characterized in terms of cellular morphology, multi-omics profile, tissue barrier function, cytokine releases, and other factors.^112–114^ The insights gathered from these simulations can elucidate complex cell–cell, tissue–tissue, and tissue–environment interactions involved in GI diseases.^112–114^

**FIG. 7.**
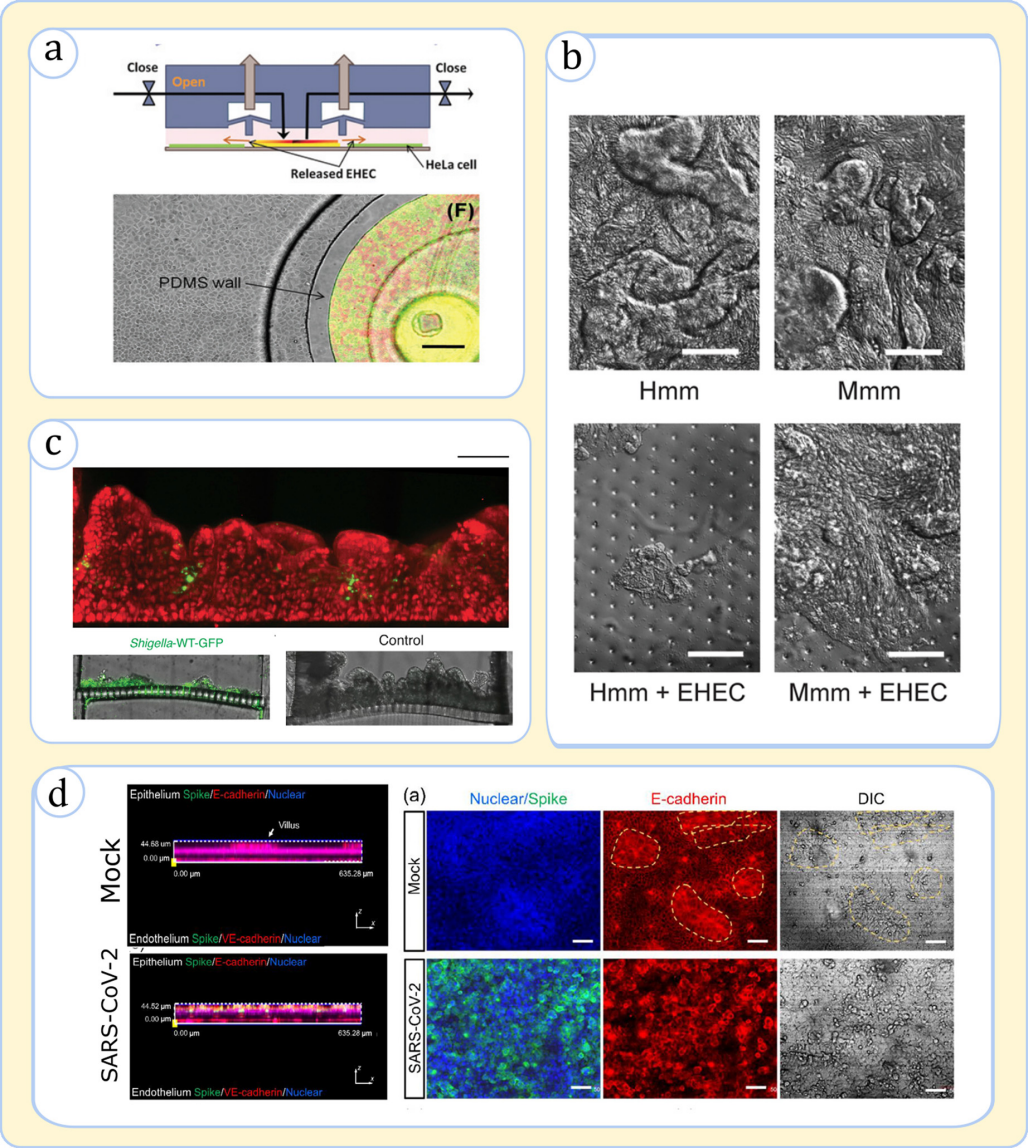
Application of gut-on-a-chip devices in modeling infections. (a) A modular microfluidic setup used to simulate EHEC bacterial infection upon interaction with commensal *E. coli* bacteria; (top) side-view sketch of the device illustrating the pneumatic actuation in the two upper channels lifts the barrier to allow mixing the bacteria with HeLa cells, the model intestinal cell used in the study; (bottom) the device top-view indicating coculture of pathogenic bacteria (red) and commensal (green) in an island before mixing with intestinal cells (grey). Used with permission from Kim *et al.*, Lab Chip **10**(1), 43 (2010). Copyright 2010 Copyright Clearance Center, Inc.^115^ (b) Simulation of EHEC infection on the intestinal chip with villi in the presence of murine and human microbiome metabolites, labeled Mmm and Hmm, respectively. The composition of Hmm causes the dissolution of villi as seen in the top-view images. Adapted with permission from Tovaglieri *et al.*, Microbiome **7**(1), 43 (2019). Copyright 2019 Authors, licensed under a Creative Commons Attribution (CC BY) license.^89^ (c) The *Shigella* infection on a chip; (top) the side-view of the epithelium (red) reveals the recessed topographies are appropriate spots for bacterial attack (green); (bottom) the side-view of epithelial cells on the membrane indicates that infection causes the destruction of villi. Adapted with permission from Grassart *et al.*, Cell Host & Microbe **26**(3), 435 (2019). Copyright 2019 Elsevier.^91^ (d) Simulation of SARS-CoV-2 infection; (left) the virus infects the epithelium while having less effect on the endothelium as seen in the side-view image; (right) the viral attack (Spike protein: green) causes villi (marked with yellow dashed lines) destruction as seen in the top-view tissue images. Adapted with permission from Guo *et al.*, Sci. Bull. **66**(8), 783 (2021). Copyright 2021 Elsevier.^118^

One well-demonstrated application of on-chip devices has been the simulation of GI bacterial infections as these devices can sustain microbial cells and human tissue cocultures.^89,91,115,116^ In one of the earliest case studies, Kim *et al.* devised a microfluidic system to model the epithelial infection caused by Enterohemorrhagic *E. coli* (EHEC) [Fig. 7(a)].^115^ The device was designed to have a modular configuration to simulate the scenarios involving the invasion of pathogenic bacteria into the gut tissue after contact with commensal bacteria. These simulations uniquely uncovered that the signaling molecules from commensal *E. coli* have a strong prohibiting effect on EHEC infection, pointing to a critical disease defense mechanism.^115^ Later, using the Emulate platform to compare EHEC infection between mice and humans, Tovaglieri *et al.* illustrated that microbiome-derived metabolites can alter the phenotype of pathogenic bacteria (e.g., chemotaxis and motility), causing tissue damage [Fig. 7(b)].^89^ In another study, Gazzaniga *et al.* used a similar platform to decipher the effect of the microbial composition against *Salmonella typhimurium.*^116^ They discovered that specific strains in the microbial composite (in their case, *Enterococcus faecium*) can elevate the host tolerance against the infection.^116^ The validity of gut-on-a-chips for infectious disease studies is also denoted by Grassart *et al.*, who demonstrated that the ability of these devices to model tissue topography, fluid flow, and peristalsis is of high significance to model the *Shigella* bacteria's invasion mechanism [Fig. 7(c)].^91^

Gut-on-a-chip designs have also exhibited noticeable applicability for modeling viral infections.^117,118^ By incorporating a dual-chamber model with vascular and luminal environments, Villenave *et al.* captured the full viral pathogenesis cycle of the coxsackievirus B1, including viral infection, replication, and propagation.^117^ The researchers notably tracked the infection path upon viral injection through the lumen or blood channel, which unveiled that final tissue damage results from an apoptotic mechanism that is triggered by the virions traveling preferentially to the apical side regardless of the point of injection. Bein *et al.* and Gou *et al.* proved that the gut-chips present unique features to model coronaviruses infections, the causative agents of diseases such as the common cold (NL63) and COVID-19 (SARS-CoV-2) [Fig. 7(d)].^118,119^ Importantly, both studies showed that angiotensin-converting enzyme 2 (ACE2) receptor, a key means of entry for coronaviruses, and protease TMPRSS2, a viral spike (S) protein priming element, are efficiently expressed in on-chip models.^118,119^ Upon exposure of gut tissue to viral loads, the chips were able to resolve the virus interactions with epithelial and endothelial cells inducing cellular damage and the loss of tight junctions [Fig. 7(d)]. These simulations have unveiled powerful means to complement animal studies considering the disparity between animal and human physiology in response to viruses.

**FIG. 8.**
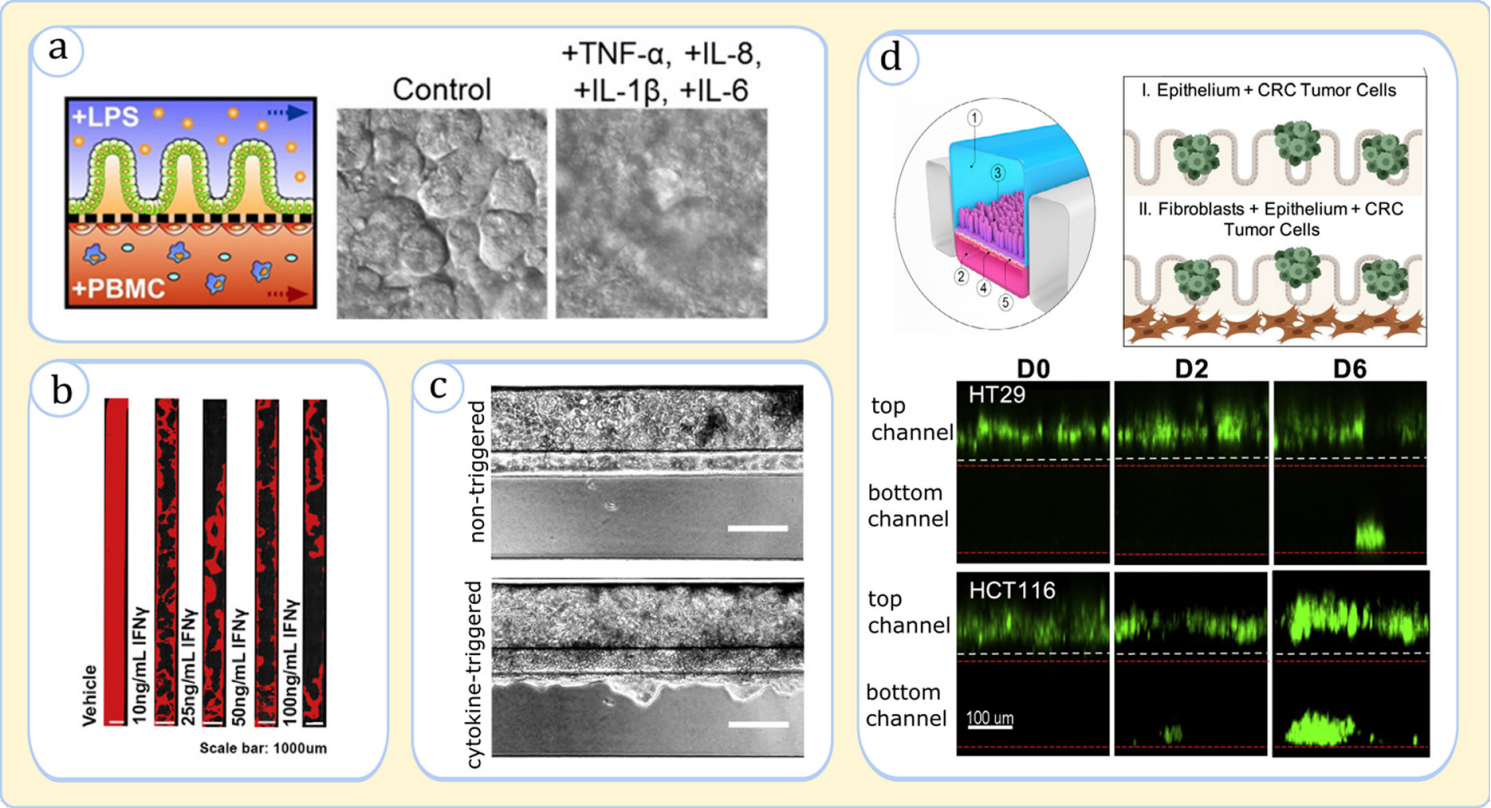
Gut-on-a-chip systems for modeling of GI diseases. (a), (b), and (c) The simulation of IBD-related scenarios, and (d) the simulation of colorectal cancer (CRC). (a) (Left) Sketch of various cells interacting in the investigated gut chip: LPS: lipopolysaccharide; (middle and right) the simultaneous exposure to LPS and PBMCs induces epithelial cells to secrete a mixture of cytokines. Images (top-view) demonstrate that the treatment of the epithelium with a particular combination of cytokines causes physical injury. Adapted with permission from Kim *et al.*, Proc. Natl. Acad. Sci. U. S. A. **113**(1), E7 (2016).^94^ (b) The decrease in epithelium confluency (DAPI-stained: red) upon the addition of IFN-γ seen across the gut chip microchannel (top-view). Adapted with permission from Apostolou *et al.*, Cell. Mol. Gastroenterol. Hepatol. **12**(5), 1719 (2021). Copyright 2021 Elsevier.^110^ (c) Subjecting the epithelium to a cytokine cocktail, IL-1β, TNF-α, and IFN-γ, for a prolonged duration (11 days) causes the invasion of the epithelium to the neighboring ECM gel (side-view). Adapted with permission from Beaurivage *et al.*, Int. J. Mol. Sci. **20**(22), 5661 (2019). Copyright 2019 Authors, licensed under a Creative Commons Attribution (CC BY) license.^120^ (d) The invasion of tumor cells HT29 and HCT116 from the top channel (lumen) into the bottom channel (vascular) across the epithelial and endothelial cells (side-view). Adapted with permission from Strelez *et al.*, iScience, **24**(5), 102509 (2021). Copyright 2021 Elsevier.^121^

Furthermore, a number of attempts have been made to examine complex GI diseases such as IBD and colorectal cancer with gut-on-a-chip models. A mimicry of gut tissue inflammation as a precursor to IBD has been presented by Kim *et al.* in a gut chip involving immune cells and bacterial endotoxins.^94^ The authors discerned a disease pathway based on the combined secretion of pro-inflammatory cytokines Interleukin 8 (IL-8), IL-6, IL-1β, and tumor necrosis factor alpha (TNF-α) by epithelial cells upon exposure to bacterial lipopolysaccharides (LPS) and PBMCs [Fig. 8(a)].^94^ The critical role of the immune function in the intestinal barrier has been reinforced in the studies of Beaurivage^120^ on the IBD and Apostolou^110^ on the leaky gut [Figs. 8(b) and 8(c)]. By screening the effect of various cytokines, these studies identified that the signaling molecules including IL-1β,^120^ TNF-α,^120^ interferon-gamma (IFN-γ),^110,120^ and IL-22^110^ are major contributors to the morphological damage and the increased permeability of epithelium. Strelez *et al.* have further illustrated the utility of gut-on-a-chip systems for colorectal cancer explorations [Fig. 8(d)].^121^ Once tumor cells were introduced into the lumen, they disrupted the metabolomic behavior of epithelium and endothelium, traversed the epithelium, and then partially dispersed in the blood circulating channel.^121^ The model was consequently deemed suitable to assess tumor invasion and identify physicochemical markers in cancer research.^121^

Overall, these findings highlight that the modular structure of gut-on-a-chips is extremely valuable in simulating gut pathophysiology by decomposing disease scenarios into less-complex case studies. These advantages as demonstrated in recent triumphs with gut-on-a-chip technologies herald a new era in modeling and comprehending complex diseases.

### B. Discovery of therapeutics

Another major application of the gut-on-a-chip is assessing drug metabolism. In the case of orally administrated medications, the drug's bioavailability can substantially subside by its traveling through multiple organs before reaching the target tissue.^122^ The intestine, in particular, has been identified as a critical determiner of drug efficiency in the first pass drug metabolism.^122^ Drug digestion in the intestine is mediated by a host of drug-metabolizing enzymes and transporter proteins secreted by enterocytes, including CYP3A4 and P-glycoprotein,^123^ and is additionally influenced by the metabolic activities of microbiota.^124^ Currently, animal and *in vitro* testing are routinely conducted to predict intestinal drug digestion; however, the former exhibit substantial differences in physiology and microbial architecture, while the latter is deficient in physiological-level drug-digesting enzymes.^41,125^

Recent evidence suggests that gut-on-a-chip devices could fill the existing voids in drug discovery by presenting multiple benefits. Data on various benchmark drugs such as verapamil and ifosfamide have shown that Caco-2 cells, once cultured in the intestine chips, expressed elevated drug-digesting enzymatic activity compared to static cultures.^83,92^ When organoids were used as the cell source, the predictability of the drug responses even further improved.^125^ Being tested against model drugs rifampicin and 1,25-dihydroxyvitamin D3, cells derived from human duodenum organoids exhibited an expression level of CYP3A4 and intestinal nuclear receptors that were akin to the physiological response.^125^ In addition, gut-on-a-chip systems have shown to be effective for assessing other physiological effects, such as the drug absorption through the intestinal barrier.^126^ This has been useful to test the efficacy of new drug formulas for oral administration as demonstrated for a modified version of the anticancer drug SN-38 (7-ethyl-10-hydroxy camptothecin).^126^

Beyond broad-scoped drug testing, a handful of studies proved that gut-on-a-chip devices could be particularly apt for developing therapies for GI disorders, as they can simulate both the disease and the treatment strategy at once. In the gut inflammation chip, for example, the administration of antibiotics into the injured tissue induced a curing effect due to the suppression of Enteroinvasive *E. Coli* (EIEC) bacteria, which corroborated clinical studies.^94^ In a different work, the assessment of the anti-inflammatory compound TPCA-1 elucidated therapeutic effects caused by the reduction of inflammatory cytokines.^127^ Another related use case has been the simulation of NL63 coronavirus infection on a gut-on-a-chip to showcase drug responses of nafamostat and remdesivir in vascular injection (blood channel) and toremifene, nelfinavir, clofazimine, and fenofibrate in oral administration (luminal channel) scenarios.^119^ The drug studies have been extended to probiotic formulations as well, which is a popular research topic among the medical commuinity.^128^ An example work has been the injection of a probiotic mixture of *Lactobacilli* and *bifidobacteria* species in the gut inflammation chip, which alleviated pathogenic infections and improved the barrier function.^94^ Nelson *et al.* characterized the effect of an engineered bacterium (synthetic biotics) for treating Phenylketonuria, a metabolic genetic disorder associated with the reduced digestion of phenylalanine in the body.^129^ The gut-on-a-chip harvested the dose-dependent metabolism of phenylalanine and yielded results aligning with *in vivo* tests.^129^ Overall, the preliminary drug discovery research based on gut-on-a-chips has been extremely promising due to the unprecedented advantages these devices offer. Accordingly, research on a broader range of drugs and therapeutics using these devices is anticipated in the near future.

### C. Clinical and industrial translation

The ability of the organ-on-a-chip to simulate diseases and drug metabolism has spurred enormous interest in commercializing these devices for clinical and industrial applications.^44^ Currently, the drive to reduce reliance on animal models is strong in biomedical, food, cosmetics, and other related industries,^39,130,131^ which has prompted regulatory bodies such as the US Food and Drug Association and the European Medicines Agency to seriously evaluate the technology^28,132^ (for a list of organ-chip manufacturing companies, refer to Singh *et al.*^130^). In the biomedical industry, in particular, the demand for a robust *in vitro* platform has been growing considering the present lengthy, complicated, and time-consuming drug discovery process.^133^ The high attrition rate of drugs is mainly attributed to false predictions in preclinical tests, driving the cost of each new medicine up to one billion dollars.^134^ Furthermore, mechanistic understanding of many diseases is still out of reach, due in part to the absence of rigorous models.^135^

Considering the promising results obtained from single organ-on-chip devices, a major initiative has focused on the development of composite devices to mimic broader physiological features, setting forth the concept of “human-on-a-chip” devices.^28,136^ In these devices, separate compartments are devoted to different organs that are connected to each other by fluidic channels or fluid movers.^137^ An example composite device is the gut-liver-on-a-chip, which, owing to the central role of the gut and liver in metabolism, has been proposed as a testing platform for drug toxicities and disease studies.^138,139^ Other multi-organ devices involve the gut combination with the kidney, brain, and skin (Table II lists example studies using these devices). Both single- and multi-organ-on-a-chip devices are envisioned to become game changers in the future, yet much work remains to be done in standardization and validation of the platform against benchmark methods.^132,134,140,141^

**TABLE II.** Select studies on multi-organ-on-a-chip platforms involving the gut.

Authors	Type of the chip	Example case study
Choe *et al.*^142^	Gut-liver	First pass metabolism of apigenin
Lee *et al.*^143^	Gut-liver	Gut absorption and liver metabolism of fatty acids
De Gregorio *et al.*^144^	Intestine-liver	First pass metabolism of ethanol and the resulting hepatic damage
Chen *et al.*^145^	Intestine-liver	Urea and albumin metabolism and CYP enzyme activity
Chen *et al.*^146^	Gut-liver	Inflammatory inter-tissue crosstalk
Maschmeyer *et al.*^147^	Liver-intestine	Repeated dose administration of troglitazone
Prot *et al.*^148^	Intestine-liver	First pass metabolism of paracetamol
Kim *et al.*^149^	Gut-brain axis	Exosomes transport across the gut barrier toward the blood-brain barrier
Lee *et al.*^150^	Gut-kidney	Antibiotic treatment effect on hemolytic uremic syndrome in Shiga-producing *E. coli* infection
Lee *et al.*^151^	Gut-skin	The effect of gut-absorbed fatty acids on the skin upon gut inflammation
Kimura *et al.*^152^	Lung-intestine-liver	The pharmacokinetics studies of three anticancer drugs-epirubicine (EPI), irinotecan (CPT-11), cyclophosphamide (CPA)
Ramme *et al.*^153^	Intestine-liver-brain-kidney	Generation of four organs from induced pluripotent stem cells
Vernetti *et al.*^154^	Intestine-liver-kidney-blood brain barrier-skeletal muscle	Absorption, metabolism, and excretion of terfenadine, trimethylamine (TMA), and vitamin D3
Imura *et al.*^155^	Intestine-liver-cancerous breast	Absorption, metabolism, and bioactivity of cyclophosphamide, epirubicin, 17-β estradiol, and soy isoflavone

## V. GUT-ON-A-CHIP LIMITATIONS AND FUTURE PERSPECTIVES

In Secs. II–IV, we described the main gut-on-a-chip technologies and elaborated on how they could be useful in pathology and drug discovery. However, just like any other biological model, gut-on-a-chips have their own shortcomings. In this section, we review some of the limitations of this platform aiming to illuminate potential future research directions.

### A. Miniaturization

Gut-on-a-chip devices are miniaturized versions of the human tissue, which cannot accurately recapitulate mechanisms that manifest at larger scales. For example, while food digestion in the intestine occurs progressively through the length of the intestine, gut-on-a-chip systems, due to small channel sizes, merely offer a short snapshot of gut metabolic and physiological processes and, thus, could overlook spatial variations of physiological parameters.

### B. Time limitations

Recent research has witnessed an increase in the lifetime of tissue and microbial cultures in the gut-on-a-chip for up to a few weeks, yet these devices cannot mimic real-life physiological time scales. This may cause inaccuracies in modeling temporal changes in microbiome composition and diseases that develop over years.^156,157^ Research into new techniques, e.g., cell culture renewal, is essential to solving this issue by improving tissue longevities in chip models.

### C. Limitations of fabrication materials

The synthesis of microfluidic scaffolds still relies heavily on the usage of artificial polymers such as PDMS. These materials, despite having practical advantages such as gas permeability and ease of fabrication, can pose technical challenges due to their absorption capacity for numerous biomolecules.^158,159^ This can disrupt the bioanalytical data derived from tissue cultures and interfere with drug studies.^158,159^ Innovative remedies for the fabrication materials and methods are required to address this issue.

### D. Diversity of cell types and complexity of the microbiome

Gut-on-a-chip devices have offered the possibility of culturing multiple cell types in one device; however, the human cellular system is still far more diverse and complex. The microbiome, for example, contains viruses, fungi, yeasts, and bacteria, and the gut mucosa is packed with fibroblasts, fibrocytes, endothelial, blood, and immune cells, which are all important in shaping the gut physiology (see Box I). One hurdle for maintaining all the cells together, however, is their different requirement of growth media and microenvironmental conditions. Extensive modularization could potentially boost cellular diversification; however, further research is needed to tackle operational issues that may arise in the resulting devices.

## VI. CONCLUSIONS

In this study, we reviewed how microfluidic devices provide an innovative approach to model gut physiology. We highlighted that gut-on-a-chip devices can tackle two critical limitations of the previous *in vivo* models simultaneously: (i) the inability to capture the function in three dimensions; and (ii) the modeling of physiochemical environmental cues which are key determinants of myriad physiological processes. So far, a variety of gut-on-a-chip schemes have been proposed. A common example of gut-on-a-chip devices is a multicompartment device where lumen and vascular systems are mimicked in juxtaposed microchannels, and a porous membrane in-between supports the growth of the gut epithelium. This platform allows for the simulation of fluid flow in the lumen and the mucosa as well as the differential hydrodynamic shear experienced by the epithelium. The peristalsis has been additionally incorporated by actuating gas pressure in adjacent ancillary microchannels or peristaltic liquid pumps. Moreover, the oxygen in each compartment has been successfully regulated through the adjustment of oxygen in the solution media and the incorporation of an anoxic enclosure. Numerous studies have demonstrated that the exposure of the cell to relevant hydrodynamics, mechanical forces, and chemical gradients results in the genetic and phenotypic expressions that mimic the *in vivo* condition, much more accurately than organoids and 2D models. Given the success of gut-on-a-chip platforms for reproducing a wide range of biological functions, these devices have been explored for a variety of clinical applications, especially in disease modeling and drug discovery. Numerous investigations have showcased that the gut-on-a-chip devices are highly meritorious for modeling a plethora of infectious and gastrointestinal diseases, in addition to testing various therapeutic interventions, including drugs and probiotics. Despite recent success, gut-on-a-chip research is still a nascent field, and extensive work remains to be conducted to overcome technical hurdles arising from fabrication limitations and operational issues resulting from miniaturization.

## Data Availability

Data sharing is not applicable to this article as no new data were created or analyzed in this study.
